# Virtual Reality Exposure Therapy for Armed Forces Veterans with Post-Traumatic Stress Disorder: A Systematic Review and Focus Group

**DOI:** 10.3390/ijerph19010464

**Published:** 2022-01-01

**Authors:** Ana Vianez, António Marques, Raquel Simões de Almeida

**Affiliations:** 1 Psychosocial Rehabilitation Laboratory, Center for Rehabilitation Research, School of Health, Polytechnic Institute of Porto, 4200-072 Porto, Portugal; acvianez@gmail.com; 2 Occupational Therapy Department, Santa Maria Health School, 4049-024 Porto, Portugal; raquel.almeida@santamariasaude.pt

**Keywords:** virtual reality, exposure therapy, post-traumatic stress disorder, military veterans

## Abstract

Virtual reality exposure therapy (VRET) is an emerging treatment for people diagnosed with Post-Traumatic Stress Disorder (PTSD) due to the limited accessibility of psychotherapies. This research aims to determine the guidelines for developing a Virtual Reality–War Scenario program for Armed Forces veterans with PTSD and encompasses two studies: Study 1, a systematic electronic database review; Study 2, a focus group of twenty-two Portuguese Armed Forces veterans. Results showed a positive impact of VRET on PTSD; however, there were no group differences in most of the studies. Further, according to veterans, new VRET programs should be combined with the traditional therapy and must consider as requirements the sense of presence, dynamic scenarios, realistic feeling, and multisensorial experience. Regardless, these findings suggest VRET as a co-creation process, which requires more controlled, personalized, and in-depth research on its clinical applicability.

## 1. Introduction

Post-traumatic stress disorder (PTSD) is a mental health problem that may occur in people who have experienced or witnessed a traumatic event such as a natural disaster, a severe accident, a terrorist act, sexual assault, or war/combat [1]. The prevalence rate of PTSD in the general adult population is currently 6.8% in the United States and 0.6–6.7% in Europe [2,3]. PTSD negatively impacts patients’ daily lives and is associated with a higher mortality risk [4]. 

According to the Diagnostic and Statistical Manual of Mental Disorders (DSM-V), PTSD includes the presence of repeated and unwanted intrusive symptoms about an event (memories, dreams, flashbacks), persistent avoidance of the stimuli associated with it (thoughts, emotions, places), negative changes in cognition and mood (distorted cognition, beliefs or expectations), and significant changes in activation and reactivity (hypervigilance, difficulty in sleeping, irritable behavior) [5]. The development depends on numerous risk factors related to individual’s psychological and cognitive vulnerabilities, poor social and family support [6,7,8], prior mental disorders, low socioeconomic status, low education level, gender (i.e., female), young age at the time of the trauma, and minority status [9]. In addition, there has been an expanding body of literature on the genetic risk factors associated with the development of PTSD [10]. It seems to be more severe and persistent when the stressful event is caused by humans and combat-trauma related [11].

In fact, research over the last decade has shown that military personnel exposed to war-zone trauma have a high risk for developing PTSD [12]. According to different studies, the prevalence rates of PTSD among soldiers and veterans can reach 30% [13]. In a 2017 study involving 5826 United States veterans, 12.9% were diagnosed with PTSD. [14] This is a strikingly high rate compared to the incidence of PTSD among the general population. In what concerns the Portuguese reality, research noted that 30% of Portuguese soldiers in the 14 years of the Portuguese colonial war had chronic PTSD [15].

In recent years, some studies have identified different types of potentially traumatizing war zone experiences that may lead to adverse psychological problems such as PTSD: committing or observing a moral injury, threats to life, atrocities or abusive violence, traumatic loss, perceived threat, and hostile environments [16]. Combat-related PTSD is usually characterized by unwanted memories, unpleasant dreams or nightmares, flashbacks, and physiological and psychological distress in response to these trauma war-zone experiences. It is also frequently associated with emotional dysregulation, social maladjustment, maladaptive cognitions, anger management difficulties, and impulsive or violent behavior [17].

Current systematic reviews with meta-analysis [18] and guidelines [19,20,21] recommend trauma-focused cognitive-behavioural therapy (CBT), cognitive processing therapy (CPT), cognitive therapy (CT), Eye Movement Desensitization and Reprocessing (EMDR), and particularly exposure therapy as effective PTSD therapies. Considering in vivo and imaginal exposure therapy inadequacy and limitations for combat-related PTSD treatment, Virtual Reality Exposure Therapy (VRET), based on the core principles of Prolonged Exposure and Cognitive Processing Therapy, has become an alternative, with promising results in this ambit [22,23,24,25]. In addition, copious evidence shows that virtual reality environments produce emotional, physiological, and behavioral responses similar to those observed in real-life situations [26].

Virtual reality exposure therapy enables the emotional engagement of patients with combat-related PTSD during exposure to a virtual war environment, bypassing avoidance symptoms and facilitating control on the therapist’s part. The sense of presence provided by an ecologically valid, highly interactive, and multisensory virtual environment facilitates the emotional processing of memories related to the traumatizing war-zone experiences [27,28,29]. This approach allows standard, gradual, and personalized exposure to the traumatic environment according to each patient’s needs and tolerance. It carries the advantages of increased control over stimuli, the possibility to repeat exposure infinitely, and the unique option to simulate environments that challenge patients according to their specific needs [30]. Several studies also point out that VRET is more effective, saving time and costs in treating various anxiety disorders, including specific PTSD. These results encourage and promote patients adherence to the VRET-based approach [31]. Nevertheless, there is limited research available about the development and efficacy of these therapies, which does not allow this innovative solution to be suggested by clinicians.

This study aims to analyse the efficacy of VRET for PTSD while determining the guidelines for designing a Virtual Reality–War Scenario program for Portuguese Armed Forces veterans diagnosed with Post-Traumatic Stress Disorder.

## 2. Methods

### 2.1. Study 1

A systematic review was conducted to gather assumptions and requirements for designing a VRET programme for Armed Forces veterans diagnosed with Post-Traumatic Stress Disorder. This systematic review followed the Preferred Reporting Items for Systematic Reviews and Meta-analysis (PRISMA) guidelines [32]. In February of 2021, searches were performed in the following electronic databases: B-on, PubMed, PTSDPubs, Clinical trials, and Cochrane Library. The search terms [“Virtual Reality”], [“PTSD”] and [“Veterans”] were used, using the term “AND” between each one, and included existing articles written in English. In addition, we used the references of papers included in our review to search for other relevant publications.

Inclusion Criteria—1. VRET was used as a therapy or as a supplement to evidence-based treatment to reduce PTSD symptoms; 2. The study focused on the efficacy of VRET to reduce PTSD symptoms; 3. PTSD symptoms were assessed with validated PTSD assessment instruments; self-reported or clinician-rated; 4. VRET minimally consisted of either an Head-mounted display—HMD that immersed a patient into a digital environment or a large projector screen that displayed the virtual environment.

**Figure 1:**
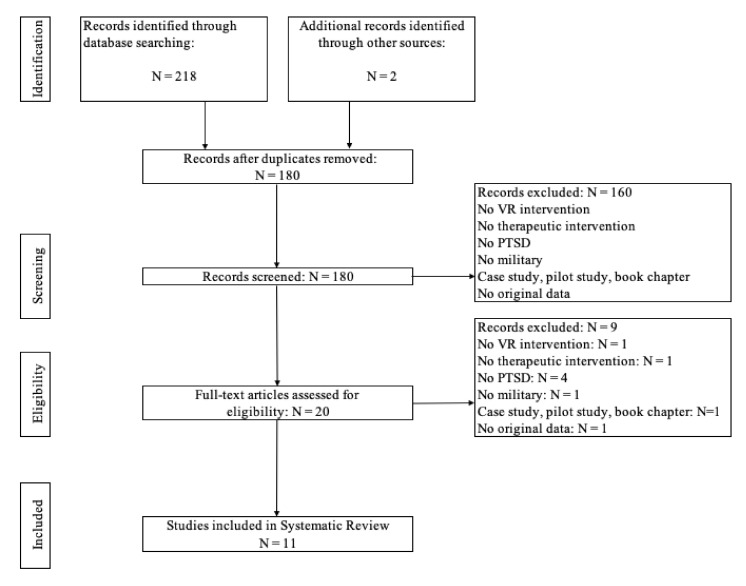
PRISMA flowchart of screening, exclusion, and inclusion criteria.

Excluded Criteria—1. Published in languages other than English; 2. Non-experimental/non-RCT studies were excluded (Figure 1).

In the research, 218 studies were identified, and the first step was to remove duplicate titles. Then, the titles and abstracts were reviewed by two independent researchers. The complete article was evaluated in case of doubt about the study’s inclusion only by its abstract. For studies that met the eligibility criteria, the full text was revised, and 11 papers were accepted for review, considering the eligibility criteria. A data-charting form was developed to determine which variables to extract, and Figure 1 outlines the study selection process. Bibliographic information, design, purpose, participants, measures, interventions, VR technology, and key findings were collected and are summarized in Table 1.

#### Quality Assessment

Each selected article was assessed using a systematic quality assessment to determine the quality of reporting and the presence of methodological bias (check the Supplementary Materials).

Studies were assessed for quality using the Downs and Black checklist. The checklist included four categories for evaluation: reporting, external validity, internal validity/bias, and internal validity/confounding. The methodological quality of all the included studies was assessed individually [33]. The score initially proposed for question 27, “Did the study have sufficient power to detect a clinically important effect where the probability value for a difference due to chance is less than 5%”? underwent a small change. Instead of the five possible scores presented by the original authors, the results were altered to 0 or 1, based on whether the authors conducted a power analysis to detect a significant clinical effect (of at least 0.80, with alpha at 0.05), with a score of 0 meaning “no” and 1 meaning “yes”. Thus, the ratings of all 28 items were either yes (=1) or no/unable to determine (=0), except for item 5, in which the scores varied as yes (=2), partially (=1), and no (=0). Classification of the final scores fell into four categories: excellent (26–28), good (20–25), fair (15–19), and poor (14 and less).

**Figure 2:**
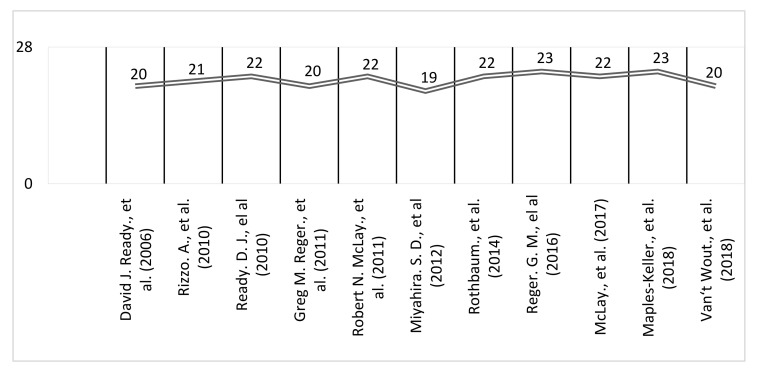
Downs and Black (1998) [33]—Checklist for assessment of the methodological quality [34,35,36,37,38,39,40,41,42,43,44].

According to the Downs and Black scores, 10 of 11 studies (N = 10/11) 90.90% had a result of good (20–25), and only one (N = 1/11) 9.09% had a result of fair (19 points) (Figure 2). 

### 2.2. Study 2

The objective of the focus group was to examine the thoughts, feelings, perceptions, and concerns about using Virtual Reality (and developing war scenarios), as well as its possible use in the treatment of armed forces veterans with war trauma. The eligible participants were Armed Forces veterans with war trauma (PTSD) involved in colonial war or peace missions. Participants were enrolled through a peer support group (Núcleo da Liga de Ex-Combatentes de Lamego). Authorization was requested from high military ranks to gain access to this group, and after the request was accepted, 22 interested participants were contacted by telephone. Participants were all war veterans who served in the Colonial War (Angola, Mozambique, and Guinea) and NATO peacekeeping missions (Timor, Iraq, Bosnia, Serbia, and Afghanistan). All were male with a mean age of 67.5 years old, (age range 55–80 years).

The effective sample received written and verbal information about the study aim and procedures. Before data collection, all participants gave written informed consent and verbal permission to record the focus group session. The focus group took place at the Special Operations Troops Center Library and by videoconference via Zoom. 

Group members were asked to introduce themselves and to state what they knew about VR. They were then asked to summarize their military histories. This introduction established the context of each person’s participation.

#### Data Collection

The focus group was conducted using semi-structured interview guidelines that included open questions about RV (war scenarios). Participants were asked to talk about: (1) What do they know about VR; (2) How they see VR as a therapeutic method; (3) VR Scenario characteristics; and (4) VR Barriers.

Some examples of semi-structured interview questions include (1) How would they see, in general, the use of VR to help deal with PTSD?; (2) What characteristics should the virtual environment have to unfold the stimulation?; (3) What narrative should it present? Where should you go? What is happening? When? With whom?; (4) Important topics: narrative; context; characters; (5) Should the scenario include different static levels?; (6) How should the instructions come up along the game? (7) How long should the game last?; (8) What precautions should we take?; (9) What are the advantages and disadvantages that are identified in its use?

The focus group was audio-recorded, and the information collected was encoded. Similar codes were grouped and organized into major themes and topics in the next step. The categories respected the criteria of relevance, homogeneity, objectivity, and purpose.

The study was approved by the Ethics Committee of the School of Health, Polytechnic of Porto (CE0064B). 

## 3. Results

### 3.1. Study 1

Table 2 summarizes the study and treatment characteristics of the eleven articles included in this review. All selected papers were quantitative and experimental studies [34,35,36,37,38,39,40,41,42,43]; the sample size was 641 subjects. The dropout rate was 160 subjects, with 481 subjects remaining in the treatments. Patients were predominantly male (96.7%). The mean age ranged from 18 to 62 years across studies. Studies included active-duty soldiers and veterans with combat-related PTSD. All selected studies for this review were carried out in the United States.

In eight of the nine studies, the reduction in PTSD symptom severity was operationalized by the Clinician-Administered PTSD Scale (two of the studies did not reveal which instrument was used). In one study, the reduction in PTSD symptom severity was operationalized by the PCL-5—PTSD checklist for DSM-5. Two studies [41,43] used the Diagnostic and Statistical Manual of Mental Disorders, 4th Edition (DSM—IV) as the instrument for PTSD diagnosis; two [35,36] used the PTSD Checklist, Military Version (PCL—M); four [34,37,42,43] used Diagnostic and Statistical Manual of Mental Disorders, 5th Edition (DSM—5); one study did not refer to the Instrument for PTSD diagnosis (32); and finally, the remaining two [39,40] used Diagnostic and Statistical Manual of Mental Disorders, 4th Edition, Text Revision (DSM—IV—TR).

In all selected studies, the therapeutic framework was prolonged exposure. PE is an exposure therapy for PTSD that received the most empirical evidence for its efficacy. It is highly effective for patients with a wide variety of traumatic experiences. In a series of randomized controlled trials, PE demonstrated major treatment effects compared to waitlist (WL) control groups and similar results compared to other active treatments, such as stress inoculation training, cognitive processing therapy, eye movement desensitization, and reprocessing [44].

Most of the studies (45.45%) used virtual Iraq/Afghanistan. The Iraq/Afghanistan VR system was developed by the Institute for Creative Technologies at the University of Southern California [45]. This tool includes a clinician’s interface that allows the therapist to customize the VR environment in real-time to match the patient’s trauma memory characteristics. As the patient recounts his/her trauma memory during imaginal exposure, the therapist fits the environment [39,40,41,42,43].

These virtual environments included comprehensive prototype scenarios of combat-related PTSD experiences, such as riding in a Humvee through a desert [7]. The software has been designed so that users can be “teleported” to specific locations within the city, based on a determination as to which components of the environment most closely match the patient’s needs relevant to their individual trauma-related experiences [35].

The head-mounted display, HMD, used for 63.63% of the studies was the eMagin z800 [35,36,39,40,41,42,43].

The sessions generally lasted between 30 and 120 min, and the average was 76.3 min per session. The number of sessions was between three and 20. Four studies included at-home in vivo exposure exercises (e.g., listening to audio recordings of each VR exposure in the memory) [34,35,41].

Six out of eleven studies (54%) explored whether the efficacy of VRET may be increased through additional medication [28,36,39,40,42,43]. Study [42] examined whether the administration of dexamethasone improved the efficacy of VRET compared to placebo treatment. Study [39] analyzed to what extent D-cycloserine and alprazolam influenced the effectiveness of VRET compared to a placebo group.

**Table 1:** Descriptive characteristics = 11 included studies.

References	Country	Instrume for PTSD Diagnosis	Primary Outcome Variable	Study Design	Sample and Trauma Type	Participants	Dropout	Intervention	Time Points of Measurements and Main Results
Ready, David J., et al. (2006) [34]	USA	DSM-IV	CAPS	Trial	Vietnam veterans with PTSD.	Total participants: N = 21 Male: 100%;	Total: N = 6;	VRET	Measurements: Pre-, post-, and 3- and 6-month follow-ups; Effect size (CAPS) All patients scored on the 3- and 6-month follow-up assessments were below their pretreatment scores (range −15 to −67%), *p* < 0.0001. Summary: All 14 patients showed reductions in PTSD symptoms compared to baseline by the 3-month follow-up assessment. These gains were maintained in 10 of the 11 patients who completed the 6-month follow-up assessment. In six of these patients, the CAPS scores continued to decline between the immediate post-treatment assessment and the 6-month assessment.
Rizzo. A., et al. (2010) [35]	USA	PCL-M	CAPS	Trial	Active duty soldiers.	Total participants: N = 20 Male: 90%; Female = 10%; Mean age = 28 years; Age range: 21–51 years;	Total: N = 6;	VRET	Measurements: Pre-, post- Effect size (CAPS) Pre-/post-PCL-M scores decreased in a statistical and clinically meaningful fashion; mean (SD) values went from 54.4 (9.7) to 35.6 (17.4). Paired pre-/post-*t*-test analysis showed these differences to be significant (t = 5.99, df = 19, *p* < 0.001). Summary: 80% of the treatment completers in this VRET sample showed both statistically and clinically meaningful reductions in PTSD, anxiety, and depression symptoms, and anecdotal evidence from patient reports suggested that they saw improvements in their everyday life situations. These improvements were also maintained at the 3-month post-treatment follow-up.
Ready. D. J., el al (2010) [34]	USA	DSM-5	CAPS	RCT	Vietnam veterans with PTSD.	Total participants: N = 11 VRET: N = 6 Male: 100%; Mean age = 57; Age range: 53–61 years; PCT: N = 5 Male: 100% Mean age = 58; Age range: 55–62 years;	Total: N = 2; VRET: N = 1; PCT: N = 1	VRET vs. PCT	Measurements: Pre-, post-, and 6-month follow-ups Effect size (CAPS) Summary: VR—31.8 (SD1⁄439.1) from pre- to post- and of 25.0 (SD 1⁄4 28.1) from pre- to follow-up, Cohen’s of 0.28 and 0.56; BDI—5.0 (SD 1⁄4 8.7) from pre- to post- and of 2.3 (SD 1⁄4 7.8) from pre- to follow-up. PCT—23.0 (SD1⁄421.9) from pre- to post- and of 13.0 (SD 1⁄4 11.3) from pre- to follow-up; Cohen of 0.0 and −0.24; BDI—of 5.0 (SD1⁄47.5) from pre- to post- and of 4.3 (SD 1⁄4 8.8) from pre- to follow-up. Combining groups—CAPS scores from pre- to post- (t 1⁄4 2.70, *p* < 0.05) and from pre- to 6-month follow-up (t1⁄42.58, *p* < 0.05). No statistically significant improvement in CAPS or BDI scores when individual treatment conditions were isolated. Summary: possible value of VRE while pointing out that the primary difficulty with further investigation of this treatment model with older veterans is participant recruitment.
Reger, Greg M., et al. (2011) [36]	USA	PCL-M	CAPS	Trial	Active duty soldiers.	Total participants: N = 32 Male: 96%; Mean age: 28.8, Gender: n.r. 75% were diagnosed with PTSD (n = 18);	Total: N = 8;	VRET	Measurements: Pre-, post- Effect size (CAPS); Pretreatment PCL-M (M = 60.92; SD = 11.03), patients receiving VRE reported a statistically significant drop in PTSD symptoms (M = 47.08; SD = 12.70), t (23) = 6.53, *p* < 0.001, d = 1.17; At post-treatment, differences on the PCL-M were no longer significant between those with PTSD (M = 49.72; SD = 13.20). Summary: Patients receiving an average of seven sessions of VRE reported statistically and clinically significant reductions in self-reported symptoms of PTSD.
McLay, Robert N., et al. (2011) [37]	USA	DSM-5	CAPS	RCT	Active Duty military personnel with combat-related PTSD.	Total participants: N = 20, VR-GET: N = 10 Male: 90% Mean age: 28.8; Gender: 22–43; TAU: N = 10 Male: 100%; Mean age: 28; Gender: 21–45;	VR-GET: N = n.r; TAU: N = n.r;	VR-GET vs. TAU	Measurements: Pre-, post-, and 10-week follow-up; Effect size (CAPS); VR-GET: N = 10, (70%) of these showed a 30% or greater improvement in the CAPS. TAU: N = 10, One (11.1%) of the 9 returning participants receiving TAU showed > 30% improvement on the CAPS. Chi-square for the treatment response comparison between VR-GET and TAU was 6.74, *p* < 0.01. With Yates correction w2 1⁄4 4.54, *p* < 0.05, relative risk was 3.21, with 95% confidence interval 1.18 to 8.72. Pre-vs. post-treatment, *p* < 0.001), but not group (*p* > 0.05). There was a significant time-by-group interaction (*p* < 0.05). There was no significant difference between VR-GET and TAU mean CAPS score before or after treatment, but there was a significant difference in the mean CAPS change score over the course of treatment (35.4 vs. 9.4, *p* < 0.05). Summary: 70% of participants who received VR-GET showed a clinically significant (>30%) improvement in their PTSD symptoms after 10 weeks of treatment. This was a significantly (*p* < 0.05) higher percentage than the 12.5% of participants who showed clinically significant responses in usual treatment.
Miyahira. S. D., et al. (2012) [38]	USA	n.r.	CAPS	RCT	Active duty service members with PTSD symptoms who participated in military operations in Iraq or Afghanistan.	Total participants: N = 99 Male: N = 94 Female: N = 5 VRE = 12 MA = 10	Total: N = 77	VRE vs. MA	Measurements: Pre-, post- Effect size (CAPS); Significant decrease over time on the CAPS Criterion C (avoidance/numbing symptoms) in the VRE group (F (1,20) = 6.03, *p* = 0.02); The VRE group scored significantly lower on the CAPS Criterion C compared to the MA group at post- procedures (F (1, 20) = 8.705, *p* = 0.008). Summary: VR exposure may be effective in reducing some PTSD symptoms in active duty service members returning from combat.
Rothbaum., et al. (2014) [39]	USA	DSM-IV-TR	CAPS	RCT	War veterans with Iraq and Afghanistan deployment; Combat-related PTSD symptoms.	Total participants: N = 156; Males = 94% Mean age: 35.1; Gender: 148; VR treatment group (VRET with DCS): n = 53; Males = 92% Mean age: 34.9; Gender: 49; Active control group (VRE with Alprazolam): n = 50; Males = 98%; Mean age: 36.2; Gender: 49 Control group (VRET with placebo): n = 53; Males = 94%; Mean age: 34.3; Gender: 50;	Total: N = 59 (37%); VR treatment group (VRET with DCS) N = 25 (47%); Active control group (VRET with Alprazolam): N = 15 (30%); Control group (VRET with placebo) N = 19 (35%);	VRET with DCS vs. VRET with Alprazolam vs. VRET with Placebo	Measurements: Pre, post, 3-, 6-, and 12-month follow-ups; Effect size: n.r. and n.a.# Summary: All groups decreased significantly on the CAPS. The effect maintained over 12 months of follow-up. At post-treatment, there was no significant difference between D-cycloserin and the placebo group for the CAPS. However, there was a significant difference favoring placebo over alprazolam regarding the CAPS at post-treatment.
Reger. G. M., et al. (2016) [40]	USA	DSM–IV-TR	CAPS	RCT	Active-duty soldiers.	Total participants: N = 162; WL: N = 53; Males = 98.15%; Mean age: 30.39 (6.45); PL: N = 51; Males = 94.44%; Mean age:30.89 (7.09); VR: N = 52; Males = 96.30%; Mean age: 29.52 (6.47);	Total: N = 6	VRE vs. PE	Measurements: Pre, midtreatment, post, 12-week and 26-week; Effect size (CAPS); VRE—Pre, 80.44 (16.23); 26-week, 53.50 (28.07); PE—Pre, 78.28 (16.35); 26-week, 38.33 (28.49); WL—Pre, 78.89 (16.87); 26-week, n.r. Summary: Results extend previous evidence supporting the efficacy of PE for active-duty military personnel and raise important questions for future research on VRE.
McLay., et al. (2017) [41]	USA	DSM-IV	CAPS	RCT	Active duty military members with past Iraq and Afghanistan deployment; Combat-related PTSD symptoms.	Total participants: N = 81; Males = 96.3%; Mean age: 32.5; Gender: 78; VR treatment group (VRET with immersive technology): n = 43; Males = 93% Mean age: 33; Gender: 40; Active control group (VRET with non-immersive technology): n = 38; Males = 100%; Mean age: 32; Gender: 38;	Total: N = 7 (8%); VR treatment group (VRET with immersive technology): N = 7 (16%); Active control group (VRET with non-immersive technology): N = 0 (0%);	VRET with immersive technology vs. VRET with non-immersive technology	Measurements: Pre, post, and 3-month follow-up Effect size (CAPS): Hedges’ gpost = −0.33# (favoring VRET with non-immersive technology) Hedges’ g3month = 0.15# (favoring VRET with immersive technology) Summary: Significant decrease on the CAPS maintained over 3-month follow-up. No significant differences between groups were found.
Maples-Keller., et al. (2018) [42]	USA	DSM-5	CAPS	RCT	War veterans and active duty personnel with past Iraq and Afghanistan deployment; Combat- related PTSD symptoms.	Total participants: N = 27; Males = 100%; Mean age: 35.4, Gender: 27 VR treatment group (VRET with dexamethasone): N = 13; Males = 100%; Mean age: n.r. Gender: 13; Active control group (VRET with placebo): N = 14; Males = 100%; Mean age: n.r. Gender: 14;	Total = 3 (12%), VR treatment group (VRET with dexamethasone): N = 0 (0%); Active control group (VRET with placebo): N = 3 (25%);	VRET with dexamethasone vs. VRET with placebo	Measurements: Pre and post Effect size (CAPS): Combined sample Cohen’s dpre-post = n.r. Summary: Significant decrease in the CAPS for post-treatment but no significant differences between groups.
Van’t Wout., et al. (2018) [43]	USA	DSM-5	PCL-5	RCT	War veterans with Iraq and Afghanistan deployment; Combat-related PTSD symptoms.	Total participants: N = 12; Males = 100%; Mean age: 40.5; Gender: 12 VR treatment group (VRET with tDCS): N = n.r. Mean age: n.r. Gender: n.r. Active control group (VRET with sham tDCS): N = n.r. Mean age: n.r. Gender: n.r.	Total = n.r. VR treatment group (VRET with tDCS) N = n.r. Active control group (VRET with sham tDCS) N = n.r.	VRET with tDCS vs. VRET with sham tDCS	Measurements: Pre, post, and 1-month follow-up Effect size (PCL-5): Hedges’ gpost = 0.20# (favoring VRET with tDCS) Cohen’s d1month = 0.37 Summary: Both groups demonstrated significant reductions in PCL scores. There were no significant differences between groups at post time measurement, but VRET with tDCS was superior to VRET sham tDCS at 1-month follow-up.

USA, United States of America; PTSD, post-traumatic stress disorder; VR, virtual reality; VRET, virtual reality exposure therapy; VR-GET, Virtual Reality-graded exposure therapy; VRE, Virtual Reality Exposure; PE, prolonged exposure; DSM-IV-TR, Diagnostic and Statistical Manual of Mental Disorders 4th Edition Text Revision; DSM-IV, Diagnostic and Statistical Manual of Mental Disorders 4th Edition; DSM-5, Diagnostic and Statistical Manual of Mental Disorders 5th Edition; CAPS, clinician-administered PTSD scale, measured via CAPS total sum score; PCL-5, PTSD checklist for DSM-5, measured via PCL-5 total sum score; RCT, randomized controlled trial; Pre, pre-treatment assessment; Post, post-treatment assessment; n.a.#, not applicable, because these studies did not report standard deviations. Instead they reported mean values and 95% confidence intervals; n.r., not reported; PCL-M, PTSD Checklist, Military Version; WL, Waitlist control; PCT, Present-centered therapy; DCS, D-cycloserine; tDCS, transcranial direct current stimulation; TAU, treatment as usual.

**Table 2:** Results of the qualitative analysis for each study.

References	Therapeutic Framework	Period of Time	Number of Sessions	Medication	Homework	Hardware	Software
David J. Ready et al. (2006) [34]	PE	Two 90-min sessions	8 to 20	n.r.	Yes; Breathing exercise for stress management and was asked to practice this exercise daily	n.r.	Virtual Vietnam
Rizzo, A et al. (2010) [35]	PE	2× weekly, 90–120-min sessions over 5 weeks	10	n.r.	Yes; First item in a hierarchical list about a traumatic event and listening to the audiotape of their exposure narrative from the most recent session	HMD—eMagin z800	Virtual Iraq
Ready, D. J., et al. (2010) [34]	PE	n.r.	10	n.r.	n.r.	n.r.	n.r.
Greg M. Reger et al. (2011) [36]	PE	90 min	3 to 12;	Yes—77% N = 16; Antidepressants—N = 12; Prazosin—N = 8; Sleep aids—N = 7; Quetiapine—N = 1; Lamotrigine—N =1; Hydroxyzine pamoate—N = 1	Yes; Listening to audio recordings of each VR exposure to the memory	HMD—eMagin z800	Virtual Iraq
Robert N. McLay et al. (2011) [37]	PE	VR-GET—1× per week for up to 10 weeks; TAU—10 weeks	VR-GET–10 TAU—14	Yes; psychotropic medications	n.r.	n.r.	n.r.
Miyahira, S. D., et al. (2012) [38]	PE	2 sessions per week for 5 weeks	10	n.r.	n.r.	n.r.	n.r.
Rothbaum et al. (2014) [39]	PE	90 min; 45 min	6; 5	Yes; D-cycloserine (50 mg); Alprazolam (0.25 mg); The placebo medication 30 min before exposure	n.r.	HMD—eMagin z800	Virtual Iraq/Afghanistan
Reger, G. M., et al. (2016) [40]	PE	90–120 min	10	Yes—n.r.	No—n.r.	HMD—eMagin z800	Virtual Iraq/Afghanistan
McLay et al. (2017) [41]	PE	90-min 30–45 min	8 to 12; 5 to 9	n.r.	Yes—Confronting real life stresses in vivo;	HMD—eMagin z800	Virtual Iraq/Afghanistan
Maples-Keller et al. (2018) [42]	PE	90-min of 7 to 12 weeks; 30–45 min	7 to 12; 6 to 11	Yes— Dexamethasone (0.5 mg) or placebo the night before virtual exposure	n.r.	HMD—eMagin z800	Virtual Iraq/ Afghanistan
Van’t Wout et al. (2018) [43]	PE	90-min of 2 weeks; 30–45 min	6; 6	Yes—n.r.	n.r.	HMD—eMagin z800	Virtual Iraq/ Afghanistan

PE, Prolonged exposure; Min, Minute; n.r., not reported; HMD, Head-mounted display.

### 3.2. Study 2

One of the authors moderated the focus groups that were conducted for about 90 min. The debate was serene, flowed naturally, and the intervention of the researcher/moderator was hardly necessary because the points that needed to be addressed were defined from the beginning. The audio-recordings were transcribed verbatim and reviewed for accuracy in transcription. Two independent researchers conducted the coding and resolved discrepancies through analysis of the raw data and input from experts on the topic. Data analysis was based on the technique of qualitative content analysis, and software webQDA was used.

Content analysis emerged on three main themes: (1) Importance of VR in PTSD, (2) VR software, (3) VR Barriers (Table 3).

VR Potential—None of the participants knew about Virtual Reality, much less that it could be used as a therapeutic tool in PTSD. After a brief explanation about VR, how it can be used, and its significant advantages, all participants agreed that it would be innovative and pertinent to technology combined with therapy. One of the former combatants said, “*the technology finally came to us*”, which shows the receptivity of this group to this therapy.

VR software—The interdisciplinary nature of VR and its evolution allow the user’s immersion, navigation, and interaction with a given platform or scenario generated by a computer to be explored by various human senses and feelings, allowing the user to exist in three dimensions: visual, sensory, and kinesthetic [46].

All participants agreed that hearing, touch, and smell stimuli should be present in a War scenario. The smell of rain and wet earth is ingrained in their memories to this day, and after so many years, it is the smell they remember most: “*The smell of heavy rain*”; “*The smell of the first rains and the earth*”. The sense of smell allows a closer approximation with reality in possible risk training sessions or psychological intervention on traumatic events [46].

Immersion was another fundamental factor and idea present in the focus group sessions. It is crucial to have the feeling of presence to create the idea of being in another place, a place full of memories, which will make the feeling of involvement. The involvement, in turn, is linked to the degree of personal motivation in a particular task or activity.

For the sense of presence to be guaranteed, it must ensure sensory fidelity, which corresponds to creating an environment with the highest possible degree of “realism”. However, making sense of presence is not limited to “showing” and “recreating” scenarios. It also implies interactivity and a psychological component [47]: “*The scenario should be dynamic and realistic*”; “*I want to feel what I felt before, look and see my memories, my pain*”.

The storyline, the quality of the narrative, and its elements are fundamental to the realism that this scenario must have. There is a military language, clothing, weapons, vehicles, fauna, and flora that will have to be present so that the illusion of presence is complete on four levels: spatial (feeling you are in a particular place); corporal (feeling you have a body); physical (being able to interact with the elements of the scenario); and social (being able to communicate with the characters in the environment) [48]—”*The bombing drove away the fauna and flora*”, “*The enemy was also the mosquitoes*”, “*There was no helmet, there were many mines*”, “*We acted in groups*”, “*I slept two years in the bush under a cloth tent*”, and “*There were no civilians, anyone who appeared after you left the barracks was considered an enemy*”.

To provide a more immersive experience, it must be possible for the individual to interact and modify the virtual environment in which the person is sensorially inserted, considering his/her emotional state. This change in the environment should be linked to the computer’s ability to detect the user inputs and instantly modify the virtual world according to its actions. This reactive capacity of the computer allows the scenes to change in response to user commands [42]: “We should have instruction before starting the immersion”, “*The evolution in the scenario should be automatic*”, and “*15 to 20 min is enough to experience the scenario*”.

It is essential that the environment created is as faithful as possible, which implies that a lot of detail matches the sensory world. These aspects are fundamental since the user takes several pieces of information from the scenario to locate himself/herself spatially.

VR Barriers—The only barrier or concern that veterans had was that they were not prepared to “enter” a war scenario again. Before they were physically and psychologically prepared to fight the enemy, they no longer had any training with these skills: “*With time, how am I going to react? Before, I was prepared, but now I’m not*”.

## 4. Discussion

### Summary of Findings

This study explores the effectiveness of VRET for PTSD in veterans and the most appropriate requirements for their implementation. According to the review, Virtual Reality for Post-Traumatic Stress Disorder treatment in former Armed Forces combatants seems beneficial. The systematic review revealed that the study of VRET protocols had a positive impact on a range of symptoms, and all treatment gains were maintained at three, six-, and 12-month follow-ups [34,35,36,37,38,39,40,41,42,43]. However, there were no group differences in most of the studies.

Most of the studies on VRET included 3–20 virtual exposure sessions, lasting 30–120 min. These studies were based on the Emotional Processing Theory (EPT). In this theory, fear is represented in memory as structures made up of associated stimulus, response, and meaning elements designed as a program to avoid or escape danger [49]. Traumatic events modify the basic beliefs of an individual since negative beliefs about the world, oneself, and others increase [49,50].

Since the key in emotion processing theory is to expose and modify its unique fear structure, discharged soldiers can control some of their destructive behaviors resulting from PTSD in a safe environment and learn how to solve these situations [51]. VR enables the patient to explore emotions while decreasing the sense of threat. It is essential to monitor anxiety levels through advanced systems and process their sensations and feelings. Any alarm should indicate to the therapist how to manage the intensity of the simulations not to cause worse harm to the patient [14]. One of the advantages of VR is to allow the therapist to control moment by moment, documenting and measuring the patient’s responses to stimuli [52].

Regarding the guidelines, the development of this VR programme should involve graphic models and narratives [14]. All of these factors were considered relevant in the focus group sessions, the narrative being one of the essential points in creating a realistic War scenario for the Armed Forces veterans. The focus group also highlighted that the specific content should be discussed in a group, then worked on with the VR resources before starting the program and not at the end. Furthermore, it is essential that the former combatants contact the VR to permit habits and that the adverse effects can be supplemented from the beginning. Another crucial factor is the individual’s initial evaluation before exposing himself/herself to the VR, to guarantee that the security conditions are reunited for their participation.

The VRET protocols varied according to medication, at-home in vivo exposure exercises, number of sessions, and period of time. Continuous monitoring is also referred to as essential. It avoids demotivation of the participants, which can cause them to give up. This monitoring may be passed on as homework (Table 2) [34,35,36,40].

The results showed that HMDs were used in seven studies in terms of the human interface. Detailed analyses revealed that 75% of the HMDs were released in 2005–2006, and the remaining 25% were released in 2012–2013. Therefore, it is necessary to deepen the effectiveness of this technology for a better understanding of its effects [53]. However, VR and its application also have limitations. The immersive nature of HMDs creates a strong presence illusion, where users perceive virtual environments (VEs) as real and not mediated through technology. The major practical issue with HMDs is that users commonly report adverse physical reactions, including headaches, nausea, dizziness, and eye strain when using them. Collectively, these symptoms represent a condition termed simulator sickness, which reportedly affects up to 80 percent of HMDs users [54,55]. Several reviews did not specify the hardware [34,37,38] or software [34,37,38] used in included studies.

Therefore, a solution that addresses discomfort experiences during a user’s first HMD exposure is essential to the continued growth and adoption of VR. This visual discomfort of VR can lead to treatment abandonment. Therefore, besides studying the efficacy of VRET, it is also crucial to investigate the safety of the treatment.

The software also imposes limitations (which we intend to address with the requirements survey conducted in the focus group). The software is often restricted to protocols created that will hinder an adequate virtual environment for the specific needs of each patient or group. Because of the pre-programmed scenarios, creating a virtual trauma-related environment that completely matches the patient’s recounting is impossible. Therefore, breaks in the sense of spatial or social presence and plausibility may occur [56].

The lack of standardized protocols is also a limitation of this therapy, which indicates the need for more research and investigation for its constitution. The publication of protocols is of vital importance to reduce costs and time that can be shared by the scientific community, in which the strengths and weaknesses are listed to avoid the elaboration of treatment and scenarios by trial and error [56]. It is also essential to choose the assessment instruments that allow measuring the effect of these interventions. Several have been pointed out in the literature, such as CAPS or PCL [57]. Further, training in VRET for therapists is essential to address the vast need for these types of interventions.

Pre-programmed virtual scenarios were used in VRET [25,34,36,39,40,41,42,43], so it may not be possible to create a trauma-related virtual environment that fully matches the patient’s narrative, which may lead to incongruity. Therefore, breaks in the sense of presence, as well as in spatial or social plausibility, can occur [58]. The choice of hardware and software depends on the type of virtual trauma intervention; the advantages and disadvantages influence the sense of presence that is supposed to impact VR scenarios’ success significantly.

The results revealed that neither spatial nor social presence was assessed in any 11 studies. All interventions with VR scenarios are based on the assumption that the sense of presence is an essential prerequisite. This aspect illustrates the need for effective research to examine whether spatial presence is a crucial mechanism for shaping the efficacy of virtual trauma interventions [59].

There was also no empirical evidence in any 11 studies on whether virtual trauma intervention was particularly effective for PTSD patients with imagination difficulties. Thus, future research is required to establish whether virtual trauma interventions are particularly effective for PTSD patients with imagination difficulties.

To sum up, these are our recommendations for developing and implementing VRET for PTSD: ensure the most immersive and sensory experience possible, engage end-users, offer a tutorial for correct operation, and rigorously evaluate the results. Moreover, the scenarios themselves must be highly customizable because, for instance, a scenario in Iraq is not at all similar to a scenario in Angola.

Although a systematic literature search was undertaken, some existing studies may have been excluded, as inclusion criteria limited papers to English. Moreover, our focus group was Portuguese Armed Forces veterans only. Therefore, caution should be taken when interpreting results, as there was some heterogeneity in the studies and samples, which limits the generalizability of the findings. Nevertheless, the data gathered could be an initial step to translate this intervention into clinical practice.

## 5. Conclusions

This study provided guidelines for developing an immersive VR program–war scenario for Armed Forces veterans diagnosed with Post-Traumatic Stress Disorder. VRET can be particularly useful in treating PTSD resistant to traditional exposure. It provides the ability to conduct extinction training/exposures for stimuli that may be too expensive or not feasible to implement in vivo, such as virtual combat situations. According to this research, new VRET programs should be combined with traditional therapy and must consider as requirements the sense of presence (spatial and/or social), dynamic scenarios, realistic feeling, multisensory experience, and should stimulate the imagination. Most of the studies on VRET included 3–20 virtual exposure sessions, lasting 30–120 min. In this co-creation process, researchers must involve end-users (mainly for the conception of narratives and content) and access all research developed on the subject to personalise the intervention and avoid inaccuracies.

We believe that the promising findings so far suggest that VRET could become a cost-efficient and effective means of providing treatment to various PTSD patient populations in the future.

## Figures and Tables

**Table 3:** Results of Study 2—Focus Group.

VR Potential	VR Software	VR Barriers
Motivation; Technology combined with traditional therapy.	Dynamic scenario; Multisensory; Realistic; Immersive; Envelopment; Stimulate the imagination	Not prepared to “enter” a war scenario again.

## Data Availability

All the data analyzed in this review are included in the present article.

## Supplementary Materials

The following are available online at https://www.mdpi.com/article/10.3390/ijerph19010464/s1, Quality Assessment.
